# Reliability of quantitative echocardiography in adult sheep and goats

**DOI:** 10.1186/1746-6148-8-181

**Published:** 2012-09-27

**Authors:** Gayle D Hallowell, Timothy J Potter, I Mark Bowen

**Affiliations:** 1 School of Veterinary Medicine and Science, University of Nottingham, Sutton Bonington, Leicestershire, LE12 5RD, UK; 2 Westpoint Veterinary Group, Dawes Farm, Bognor Road, Warnham, West Sussex, RH12 3SH, UK

**Keywords:** Ovine, Caprine, Cardiac, Heart, Measurements, Ultrasonography

## Abstract

**Background:**

Echocardiography is a non-invasive method for assessment of the ovine and caprine heart. Complete reference ranges for cardiac dimensions and time indices for both species are not currently available and reliability of these measurements has not been evaluated. The objectives for this study are to report reliability, normal cardiac dimensions and time indices in a large group of adult sheep and goats.

Fifty-one adult sheep and forty adult goats were recruited. Full echocardiographic examinations were performed in the standing unsedated animal. All animals underwent echocardiography four times in a 72-hour period. Echocardiography was performed three times by one author and once by another. Images were stored and measured offline. Technique and measurement repeatability and reproducibility and any differences due to animal or day were evaluated. Reference ranges (mean ± 2 standard deviations) were calculated for both species.

**Results:**

Majority of the images obtained were of good to excellent quality. Image acquisition was straightforward with 5.4% of animals demonstrating a small scanning window. Reliability was excellent for majority of dimensions and time indices. There was less variation in repeatability when compared with reproducibility and differences were greater for technique than for measurements. Dimensions that were less reliable included those for right ventricular diameter and left ventricular free wall. There were many differences in cardiac dimensions between sheep and goats.

**Conclusions:**

This study has demonstrated that specific reference ranges are required for these two species. Repeatability and reproducibility were excellent for the majority of cardiac dimensions and time indices suggesting that this technique is reliable and valuable for examination of clinical cases over time and for longitudinal research studies.

## Background

Echocardiography is a non-invasive method for assessment of the ovine and caprine heart. However, it is a technique that has been utilised more frequently in the assessment of clinical disease in small animals and horses for evaluation of changes in wall thickness, chamber size and valvular appearance and function. For measurements to be accurate and reliable, images must be taken from correctly orientated imaging planes in relation to internal landmarks [1]. For longitudinal studies to be undertaken and individual cases to be evaluated by different clinicians, knowledge of the reliability of these measurements is required. To the authors’ knowledge, no studies have been undertaken evaluating technique and measurement reliability of echocardiographic parameters in sheep and goats. There are no studies reporting a full set of cardiac dimensions and time indices in adult sheep, although one study has reported normal M-mode measurements in adult sheep [2], several papers have addressed cardiac dimensions and time indices in fetal and juvenile lambs [3-5] and others have investigated various aspects of cardiac function relating to valvular implants and regurgitation in sheep for experimental purposes and for application to human patients [6]. There have been two studies in goats [7,8], one reporting a range of cardiac dimensions, but no time indices [8] and the other reporting a subset of cardiac measurements at different stages of the reproductive cycle [7].

Sheep and goats are infrequently clinically diagnosed with structural cardiac abnormalities. This may be due to these species being relatively resistant to cardiac disease or because these animals are rarely presented for detailed medical evaluation. Descriptions of endocarditis in small ruminants have not been reported. Myocarditis due to bacterial (e.g. *Clostridial spp*. and *Mycobacterium spp*.), viral (e.g. foot and mouth disease), parasitic (e.g. toxoplasmosis or sarcocystosis) or toxic causes (e.g. monensin, gossypol, *Cassia occidentalis*, *Phalaris* spp, oleander) could be seen in small ruminants; reports are however lacking [9]. There are only two case reports in the literature that describe congenital cardiac abnormalities in goats; three cases of ventricular septal defects in related Saanens [10] and an Ebstein’s anomaly identified using echocardiography in a pygmy goat [11]. There are reports in sheep of ventricular septal defects [12], tetralogy of Fallot [13], patent ductus arteriosus [14] and other rarer congenital cardiac abnormalities [12,13] which were all identified at post-mortem examination. However the interpretation of any echocardiographic changes seen alongside such abnormalities can be problematic (unless end-stage disease present) if normal cardiac dimensions and the reliability of these measurements are unknown.

The aims of this study are to report reliability, normal cardiac chamber dimensions and time indices in a large group of adult sheep and goats. Methods for obtaining echocardiograms based on external anatomical landmarks have been described [15,16] and adapted for obtaining quantitative measurements based upon internal landmarks by other authors [17,18] in horses previously. These guidelines have been used in this study in order to ensure consistent images and thus measurements are obtained.

## Methods

### Study animals

Fifty-one adult sheep and forty adult goats were recruited. Eight of the sheep and one goat were the property of the Royal Veterinary College; the remaining forty-three sheep and thirty-nine goats were the property of private owners. All animals received a full clinical examination and a base-apex electrocardiogram (ECG) was recorded at rest. The animals were only included in the study if they were reported to have been in good health in the previous four weeks prior to echocardiographic examination. Animals were excluded from the study if a cardiac murmur was detected on auscultation or if any dysrhythmia was present on the base-apex electrocardiogram, including sinus tachycardia (>100 beats per minute). Any animals with structural valvular disease identified on echocardiographic analysis were excluded from the study. This study was approved by the Royal Veterinary College Ethics and Welfare Committee.

### Study protocol

This was a prospective experimental study. All animals were weighed and scored for body condition (BCS) using a 5-point scale for sheep [19] and 9-point scale for goats [20] prior to echocardiographic examination. Age was obtained from the animals’ records. Prior to restraint, the animals were allowed to acclimatize for 10 minutes in the environment in which the examination was to be performed. The sheep and goats were minimally restrained with a seated holder restraining the animal from the side with an arm lightly supporting their neck. A small square area on the left and right hemithorax just behind the elbows was clipped and surgical spirit was used to clean the skin. Ultrasound coupling gel^a^ was then applied and allowed to soak into the skin for a minimum of five minutes. An electrocardiogram was concurrently recorded whilst echocardiography was being performed. The positive and negative leads were placed in the right and left axillae and the ground lead attached to the skin cranial to the scapula. The leads were placed in these locations in order that an adequate trace was acquired without interfering with ultrasound probe and positioning.

### Echocardiographic protocol

**Figure 1:**
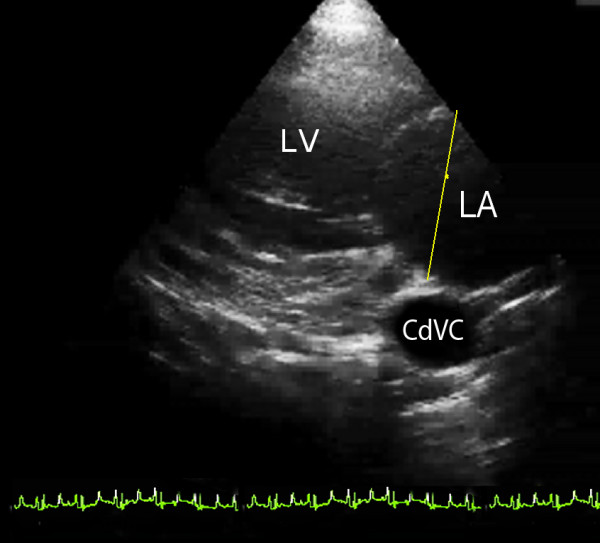
**Left parasternal caudal long axis view of the mitral valve, which is in the centre of the image.** The left atrium (LA) is to the right of the image and the left ventricle (LV) to the left. The yellow line represents the position that the left atrium was measured. The caudal vena cava (CdVC) is used as a landmark for consistently obtaining this view.

All echocardiographic examinations were performed in the standing unsedated animal. All animals underwent echocardiography four times in a 72-hour period. They were scanned three times by one author (GH) and once by another. In order to keep the animals as relaxed as possible they were allowed to stand in a position that was comfortable and were only encouraged to abduct their forelimbs when absolutely necessary. Six two-dimensional (2-D) parasternal images were obtained from the right and three 2-D parasternal images from the left as described for horses previously [17]. In addition three M-mode images were obtained from the right side and colour-flow Doppler (CFD) was used to interrogate all valves. A 5 MHz phased-array transducer attached to one of two ultrasound machines^b,c^ was used to acquire the images. Coupling gel was applied to the transducer, and this was applied to the skin approximately 2–3 cm dorsal to the olecranon in the 4^th^ and 5^th^ right intercostal spaces (ICS) and the 4^th^ and 5^th^ left ICS. Depth and gain controls were adjusted to optimise the image appearance as required. The images that were obtained included right parasternal cranial long axis view of the right ventricular outflow tract, right parasternal long axis views of the left ventricular outflow tract and the right parasternal long axis view of the left ventricle. Right parasternal short axis views were obtained of the left ventricle, mitral valve and aortic valve and M-mode images were obtained of the aortic valve, mitral valve and left ventricle from these views. Left parasternal caudal long axis view of the mitral valve and left atrium was obtained (Figure 1) as were left parasternal cranial long axis view of the aortic valve and left ventricle and left parasternal cranial long axis view of the right ventricular outflow tract. The ICS and probe orientation used to obtain each image was recorded as was the ease of acquisition and any noteworthy findings were recorded at the end of the examination.

### Data collection and statistical methods

All images were stored and analysed at a later date using the dedicated software of the appropriate ultrasound machine^b,c^. A minimum of five cardiac cycles were saved. Measurements were performed using the electronic callipers within the analysis system using the leading-edge to leading-edge method technique as outlined by the American Society of Echocardiography [21].

Measurements recorded from 2-D images included pulmonary artery diameter (PA) in diastole, aortic diameter (Ao) in diastole, aortic sinus diameter (AoS) in diastole, left atrial diameter (LAD) in systole and cross-sectional diameter of the aorta (Ao-cs) in diastole. Measurements recorded from M-mode measurements in both systole and diastole included right ventricular diameter (RVDs and RVDd), inter-ventricular septum thickness (IVSs and IVSd), left ventricular diameter (LVDs and LVDd) and left ventricular free-wall (FWs and FWd). Other M-mode measurements recorded included ejection time (ET) and pre-ejection period (PEP) and E-point to septal separation (EPSS). Calculated measurements include the ratio of the pulmonary artery diameter to the aortic diameter (PA:Ao), fractional shortening (FS = 100*[LVDd-LVDs]/LVDd) and left ventricular ejection fraction (LVEF). The latter was calculated with the integrated calculation program ([(A-B)*100/A] where A = (7*D_1_^3^)/(2.4*D_1_) and,B = (7*D_2_^3^)/(2.4*D_2_). D_1_ is the left ventricular diastolic diameter (cm) and D_2_ is the left ventricular systolic diameter (cm)) of the ultrasound software^b,c^. Measurements were only included in the analysis if they were obtained from images deemed of good to excellent quality. Excellent quality images allowed clear, easy visualisation of all structures of interest, good quality images were as for excellent, but spontaneous contrast or lung obscured certain cardiac structures for some of the cardiac cycle, images were deemed to be of average quality when there was poor definition of some of the structures but measurements could still be performed and images were deemed poor was when there were several structures that could not be easily visualised for much of the cardiac cycle. Heart rate was recorded from the ECG that was attached to the ultrasound machine.

To assess technique repeatability, one echocardiographer undertook three examinations of the same animal daily for three days and measurements were performed by one observer. To assess technique reproducibility, two echocardiographers each performed one examination per animal on the same day and measurements were performed by one observer. In order to assess measurement repeatability, each measurement was repeated blindly on three occasions at least one week apart on all echocardiograms by one observer. In addition a second observer measured a subset of echocardiograms on three occasions at least one week apart providing an indicator of repeatability for each observer and overall. To assess reproducibility, measurements and time indices were compared for each animal across operators. One operator performed measurements and time indices on all echocardiograms and the second operator undertook the same procedure in a subset of echocardiograms.

To identify other potential factors affecting reliability, echocardiographic observations, measurements and time indices were undertaken from cineloops obtained on the same and consecutive days for each animal and measurements within and between animal and species were also evaluated for differences.

#### Statistical analysis

Data were presented as the mean ± standard deviation (SD) from both observers’ measurements. Reference ranges were also calculated (mean ± 2SD). Measurements between species were compared using a Student’s *T*-test. Data was then corrected for bodyweight; each individual measurement was divided by the individual animals’ bodyweight and also using Ao. Reliability was assessed by demonstrating concordance, using percentage agreement and statistical measures of agreement (intra-class correlation coefficient (ICC) and Cohen’s Kappa). Concordance (ICC and Cohen’s Kappa) was considered excellent when greater than 0.8, good when 0.6-0.8, average when 0.4-0.6 and poor when less than 0.4 [22]. Reliability was assessed using analysis of variance (ANOVA), repeated measures ANOVA and paired Student’s *T*-test. Comparisons between this study and others were made using independent means *T*-test. All statistical analyses were undertaken using commercial statistical packages^d,e^. Significance was assumed when p < 0.05.

## Results

### Animals

Fifty-one sheep (Suffolk cross; eleven rams and forty non-pregnant, non-lactating ewes) and forty goats (7 Golden Guernsey and 33 Saanen; thirty-five non-pregnant, non-lactating females and five males) were used in this study. The sheep were aged 2–4 years and weighed 74 ± 13Kg with a range of BCS from 2.0-3.5 out of 5. The goats were aged 18 months to 3 years and weighed 48 ± 10Kg with a range of BCS from 4–6 out of 9. No animals were tachycardic at the beginning or during any echocardiographic examination. No murmurs were auscultated in any of the animals. Two goats and one sheep had marked sinus dysrhythmia at rest, which was confirmed with electrocardiography. All other electrocardiograms demonstrated sinus rhythm.

Heart rates of the sheep during the examination were 84.7 ± 7.5 beats per minute (bpm) and in goats was 90.1 ± 6.3 bpm. Two goats had mild tricuspid regurgitation and two sheep mild mitral regurgitation diagnosed using CFD; these were not associated with audible murmurs or any notable structural valvular abnormalities and thus the animals were not excluded from the study.

### Image generation and appearance

The main minor limitation encountered with image acquisition involved the small scanning window in two of the goats and three of the sheep (5.4% of all animals scanned) due to obscurement of the heart by the pleural surface. This was overcome on re-examination, although image quality in these animals was deemed adequate, and thus they were not included in the final analysis. The animals were often unwilling to stand with the right forelimb protracted cranially, but were much more accepting of gentle persuasion to abduct this limb. This was most frequently required in order to obtain the right parasternal cranial long axis view of the right ventricular outflow tract and for the right parasternal short axis views.

Nine 2-D image loops (short and long-axis) and three M-modes were obtained in all ninety-one animals. A summary of specific findings for each view is listed in Table 1 along with modifications of probe orientation compared to previous descriptions in horses [15,16,18].

**Table 1:** Describing how images were obtained and what structures were visualised

**View**	**Intercostal space image obtainedfrom**	**Probe orientation**	**Structures visualised**	**Comments**
Right parasternal cranial long axis view	RICS4	0° rotation	PA, PV, RVOT, and TV	Adequate quality in 5.4% due to small scanning window
Right parasternal caudal long-axis view of the LVOT	RICS4	Transducer rotated between 0 and 30°	Ao, AoV, LVOT and RV	TV only visible in 78.4% of the sheep and 70% of the goats
Right parasternal long axis view of the LV	RICS4 - 88.2% sheep and 70% goats RICS5 – 11.8% sheep and 30% goats	Caudal with 0° rotation	LV, MV, RV and TV	Could not visualise entire LA and RA
Right parasternal caudal short axis view of the LV	RICS4 2 cm above point of olecranon	90° rotation	LV, papillary muscles in LV, RV and TV	Symmetrical views in 91.2% of animals
Right parasternal caudal short axis view of the MV	RICS4 2 cm above point of olecranon	Caudo-dorsally with 90° rotation	MV and RV	Symmetrical views were obtained in 94.5% of animals Image occasionally obscured by pleural surface
Right parasternal caudal short axis view of AoV	RICS4 2 cm above point of olecranon	Dorsally oriented with 90° rotation	Ao, MV, RV and TV	Pleural surface visible in 71.4% of images
Left parasternal caudal long axis view (Figure 1)	LICS5 in 76.9% and LICS4 in 13.1% 2-3 cm above the olecranon	Caudally oriented with 0° rotation	CdVC, LA, LV, and MV	Spherical CdVC used as landmark in the far field
Left parasternal cranial long axis view	LICS4	Cranio-dorsally oriented with 0 to -20° rotation	Ao, AoV CdVC, and LVOT	
Left parasternal cranial long-axis view of the RVOT	LICS4 in 56% and LICS3 in 44%	Cranio-dorsally, with 0° rotation	Ao, PA, PV RA, RVOT and TV	

Table 1 Descriptions of how standard views described for horses [17,18] were adapted to obtain equivalent images in sheep and goats, which intercostal space they were obtained from, what structures were visible and any other important notes. Abbreviations: Ao: aorta; AoV: aortic valve; CdVC: caudal vena cava; LA: left atrium; LICS: left intercostal space; LVOT: left ventricular outflow tract; LV: left ventricle; MV: mitral valve; PA: pulmonary artery; PV: pulmonary valve; RA: right atrium; RICS: right intercostal space; RV: right ventricle; RVOT: right ventricular outflow tract; TV: tricuspid valve.

Except for those 5.4% of animals that had a small scanning window, the images were relatively straightforward to obtain. Pleural surface interference was a minor problem in the majority of the animals; for the short axis views of the mitral and aortic valves, the probe had to be moved ventrally down the chest wall and angled dorsally. No problems were encountered regarding ICS width and image generation. Many of the animals were unwilling to stand with the limb moved cranially, but were much more tolerant of the limb being gently abducted.

Overall 819 cineloops were obtained. Fifty-nine cineloops (7.2%) from 21 animals were deemed of average quality, 254 (31%) cineloops were deemed good quality and 506 (61.8%) were deemed of excellent quality. There was no correlation between image quality and bodyweight or BCS in these animals (p > 0.24).

### Echocardiography

Reference ranges and mean ± SD for cardiac dimensions and time indices are displayed in Table 2 for sheep and Table 3 for goats. Colour-flow Doppler revealed a relatively low proportion of animals with physiological regurgitation and there was no difference in prevalence between sheep and goats. 9.9% of animals had pulmonary regurgitation, 4.4% had physiological aortic regurgitation and 5.5% had tricuspid regurgitation. No animals demonstrated evidence of mitral regurgitation.

**Table 2:** Cardiac dimensions, time indices, technique and measurement reliability from 51 adult healthy sheep

**Parameter**	**Reference range (mean±2SD)**	**Mean±SD**	**Technique repeatability**		**Technique reproducibility**			**Measurement repeatability**		**Measurement reproducibility**		
			**Max% difference**	**ICC**	**Max% difference**	**ICC**	**Kappa**	**Max% difference**	**ICC**	**Max% difference**	**ICC**	**Kappa**
PA (cm) + *	1.96-2.80	2.38±0.21	2.5	0.92	3.8	0.88	0.87	2.3	0.94	2.8	0.91	0.89
Ao (cm) + *	2.24-3.24	2.74±0.25	2.9	0.90	4.2	0.86	0.85	2.6	0.92	3.1	0.88	0.89
PA: Ao	0.77-1.01	0.89±0.06										
AoS (cm)	2.58-3.82	3.20±0.31	3.2	0.90	3.0	0.90	0.88	2.8	0.92	3.2	0.89	0.87
Ao-cs (cm)	2.49-3.69	3.09±0.30	2.7	0.93	3.6	0.88	0.87	2.4	0.94	2.8	0.90	0.90
LAD (cm)+	2.83-6.35	4.59±0.88	2.6	0.91	4.1	0.84	0.82	2.2	0.94	2.7	0.89	0.88
ET (sec)	0.11-0.27	0.19±0.04	3.1	0.90	3.3	0.89	0.87	2.4	0.93	2.6	0.92	0.91
PEP (ms)	0.01-0.05	0.03±0.01	2.8	0.88	3.0	0.87	0.96	2.6	0.92	2.9	0.90	0.92
EPSS (cm)	0.24-0.60	0.42±0.09	3.3	0.85	3.7	0.82	0.80	2.7	0.90	3.0	0.92	0.86
RVDd (cm)	0.70-2.14	1.42±0.36	5.9	0.66	7.2	0.51	0.56	3.4	0.84	4.4	0.74	0.64
RVDs (cm)	0.56-1.12	0.84±0.14	6.2	0.72	6.6	0.57	0.59	3.6	0.82	4.1	0.72	0.66
IVSd (cm) + *	0.89-1.49	1.19±0.15	3.8	0.84	3.6	0.82	0.84	2.8	0.91	3.2	0.87	0.85
IVSs (cm)+	1.19-1.91	1.55±0.18	4.2	0.82	3.8	0.82	0.84	3.0	0.90	3.5	0.88	0.88
LVDd (cm)+	3.34-5.50	4.42±0.54	2.4	0.91	2.7	0.90	0.89	2.2	0.93	2.5	0.90	0.88
LVDs (cm)*	1.92-3.32	2.62±0.35	2.8	0.92	3.2	0.90	0.88	2.5	0.94	2.6	0.92	0.90
FWd (cm) + *	0.71-1.27	0.99±0.14	4.8	0.82	5.7	0.76	0.74	3.4	0.84	3.9	0.79	0.78
FWs (cm)*	0.94-2.06	1.50±0.28	5.2	0.81	6.3	0.74	0.73	3.6	0.86	4.1	0.81	0.80
FS (%)	30.6-49.8	40.2±4.8										
LVEF (%)	67.1-86.7	76.9±4.9										
HR (bpm)	73.5-95.1	84.3±5.4										

Table 2 Reference ranges (mean± 2 standard deviations) and mean ± standard deviation for cardiac dimensions, time indices and heart rate from 51 adult healthy sheep. Technique and measurement repeatability and reproducibility are also displayed and assessed using intra-class correlation coefficients (ICC) and maximal differences between measures. Cohen’s Kappa was additionally used to assess reproducibility. * shows significant species differences and ^+^ shows significant differences when corrected for bodyweight; p < 0.05. **Abbreviations:** Ao: aorta; Ao-cs: aorta measured from a short axis view (cross-section); AoS: aortic sinus; EPSS: e-point to septal separation; ET: ejection time; FS: fractional shortening; FWd: left ventricular free wall measured in diastole; FWs: left ventricular free wall measured in systole; HR: heart rate; IVSd: inter-ventricular septum measured in diastole; IVSs: inter-ventricular septum measured in systole; LAD: left atrial diameter measured end systole; LVDd: left ventricular diameter measured in diastole; LVDs: left ventricular diameter measured in systole; LVEF: left ventricular ejection fraction; PA: pulmonary artery PA: Ao: ratio of pulmonary artery to aorta; PEP: pre-ejection period; RVDd: right ventricular diameter measured in diastole; RVDs: right ventricular diameter measured in systole.

**Table 3:** Cardiac dimensions, time indices, technique and measurement reliability from 40 adult healthy goats

**Parameter**	**Mean±SD**	**Reference range (mean±2SD)**	**Technique repeatability**		**Technique reproducibility**			**Measurement repeatability**		**Measurement reproducibility**		
			**Max% difference**	**ICC**	**Max% difference**	**ICC**	**Kappa**	**Max% difference**	**ICC**	**Max% difference**	**ICC**	**Kappa**
PA (cm) + *	1.93±0.28	1.37-2.49	2.8	0.91	3.6	0.87	0.88	2.1	0.95	2.6	0.91	0.90
Ao (cm) + *	2.18±0.26	1.66-2.70	3.1	0.89	4.0	0.82	0.83	2.7	0.92	3.3	0.89	0.88
PA: Ao	0.93±0.04	0.85-1.01										
AoS (cm)	2.64±0.30	2.04-3.24	2.8	0.90	3.2	0.88	0.87	2.4	0.94	3.1	0.85	0.86
Ao-cs (cm)	2.42±0.28	1.86-2.98	3.3	0.86	3.8	0.81	0.82	2.6	0.92	2.9	0.90	0.90
LAD (cm)+	4.06±0.54	2.98-5.14	3.2	0.86	4.3	0.81	0.80	2.5	0.93	3.6	0.82	0.81
ET (sec)	0.18±0.05	0.08-0.28	3.3	0.88	3.7	0.84	0.83	2.7	0.91	3.2	0.87	0.88
PEP (ms)	0.03±0.01	0.01-0.05	3.2	0.85	3.4	0.87	0.88	2.3	0.95	2.6	0.89	0.90
EPSS (cm)	0.37±0.09	0.19-0.55	3.0	0.88	3.5	0.86	0.84	2.5	0.93	3.2	0.85	0.84
RVDd (cm)	0.99±0.32	0.35-1.63	6.3	0.64	6.7	0.55	0.58	3.9	0.81	4.5	0.76	0.70
RVDs (cm)	0.67±0.28	0.15-1.19	6.5	0.68	7.1	0.52	0.54	3.8	0.81	4.3	0.76	0.72
IVSd (cm) + *	0.98±0.21	0.56-1.4	3.4	0.88	3.8	0.81	0.82	2.9	0.89	3.1	0.85	0.83
IVSs (cm)+	1.32±0.32	0.68-1.96	3.6	0.86	4.1	0.80	0.84	3.1	0.86	3.2	0.83	0.82
LVDd (cm)+	3.74±0.48	2.78-4.70	2.2	0.96	2.6	0.92	0.90	1.8	0.97	2.3	0.92	0.91
LVDs (cm)*	2.11±0.31	1.49-2.73	2.4	0.94	3.0	0.88	0.86	2.1	0.96	2.5	0.92	0.92
FWd (cm) + *	0.79±0.07	0.65-0.93	3.8	0.81	4.6	0.75	0.73	3.2	0.86	3.6	0.78	0.79
FWs (cm)*	1.22±0.19	0.84-1.60	4.2	0.80	5.2	0.73	0.71	3.4	0.84	3.8	0.80	0.81
FS (%)	45.2±5.9	33.4-57.0										
LVEF (%)	73.6±5.4	62.8-84.4										
HR (bpm)	87.1±3.2	80.7-93.5										

Table 3 Reference ranges (mean± 2 standard deviations) and mean ± standard deviation for cardiac dimensions, time indices and heart rate from 40 adult healthy goats. Technique and measurement repeatability and reproducibility are also displayed and assessed using intra-class correlation coefficients (ICC) and maximal differences between measures. Cohen’s Kappa was additionally used to assess reproducibility. * shows significant species differences and ^+^ shows significant differences when corrected for bodyweight; p < 0.05. **Abbreviations:** Ao: aorta; Ao-cs: aorta measured from a short axis view (cross-section); AoS: aortic sinus; EPSS: e-point to septal separation; ET: ejection time; FS: fractional shortening; FWd: left ventricular free wall measured in diastole; FWs: left ventricular free wall measured in systole; HR: heart rate; IVSd: inter-ventricular septum measured in diastole; IVSs: inter-ventricular septum measured in systole; LAD: left atrial diameter measured end systole; LVDd: left ventricular diameter measured in diastole; LVDs: left ventricular diameter measured in systole; LVEF: left ventricular ejection fraction; PA: pulmonary artery PA: Ao: ratio of pulmonary artery to aorta; PEP: pre-ejection period; RVDd: right ventricular diameter measured in diastole; RVDs: right ventricular diameter measured in systole.

### Comparison to previous measurements

Table 4 demonstrates comparisons between this study and those previously published [2,7,8]. Significant differences included PA, IVSd, LVDd and LVDs, LVEF and bodyweight in goats [8] and RVDd and LVDd and LVDs in sheep [2].

**Table 4:** **Cardiac dimensions and time indices from this study compared with previous ones**[2,7,8]

**Parameter**	**Goats – current study**	**Goats – Leroux etal. 2012**	**Goats – Olsson etal. 2001**	**Sheep – current study**	**Sheep – Moses andRoss 1987**
PA (cm)	1.93±0.28	**2.45±0.07**	NR	2.38±0.21	NR
Ao (cm)	2.18±0.26	2.03±0.10	NR	2.74±0.25	NR
PA: Ao	0.93±0.04	NR	NR	0.89±0.06	NR
AoS (cm)	2.64±0.30	2.83±0.10	NR	3.20±0.31	NR
Ao-cs (cm)	2.42±0.28	NR	NR	3.09±0.30	NR
LAD (cm)	4.06±0.54	NR	NR	4.59±0.84	NR
ET (sec)	0.18±0.05	NR	NR	0.19±0.04	NR
PEP (ms)	0.03±0.01	NR	NR	0.03±0.01	NR
EPSS (cm)	0.37±0.09	NR	NR	0.42±0.09	NR
RVDd (cm)	0.99±0.32	1.21±0.21	NR	1.42±0.36	**2.03±0.56**
RVDs (cm)	0.67±0.28	NR	NR	0.84±0.14	NR
IVSd (cm)	0.98±0.21	0.88±0.07	NR	1.19±0.15	0.94±0.17
IVSs (cm)	1.32±0.32	1.48±0.11	NR	1.55±0.18	1.41±0.22
LVDd (cm)	3.74±0.48	**4.81±0.37**	4.06±0.1	4.42±0.54	**5.17±0.74**
LVDs (cm)	2.11±0.31	**2.74±0.24**	2.4±0.8	2.62±0.35	**3.23±0.46**
FWd (cm)	0.79±0.07	0.94±0.09	0.68±0.03	0.99±0.14	0.89±0.20
FWs (cm)	1.22±0.19	1.53±0.09	1.29±0.06	1.50±0.28	1.53±0.11
FS (%)	45.2±5.9	43.0±3.11	40.6±7.9	40.2±4.8	37.2±5.7
LVEF (%)	73.6±5.4	**73.9±3.58**	NR	76.9±4.9	NR
HR (bpm)	87.1±3.2	95.8±16.3	**107±9**	84.3±5.4	96.1±21.6
Weight (kg)	48±10	**66±9**	51±2	74±13	74±11

Table 4 is displaying mean ± standard deviation for cardiac dimensions and time indices in sheep and goats obtained in this study. These have then been compared with dimensions reported from two previous studies in goats [7,8] and one in sheep [2]. Figures displayed in bold have been shown to be statistically different (p < 0.05) from values obtained in the current study when compared using independent means *T*-test. **Abbreviations:** Ao: aorta; Ao-cs: aorta measured from a short axis view (cross-section); AoS: aortic sinus; EPSS: E-point to septal separation; ET: ejection time; FS: fractional shortening; FWd: left ventricular free wall measured in diastole; FWs: left ventricular free wall measured in systole; HR: heart rate; IVSd: inter-ventricular septum measured in diastole; IVSs: inter-ventricular septum measured in systole; LAD: left atrial diameter measured end systole; LVDd: left ventricular diameter measured in diastole; LVDs: left ventricular diameter measured in systole; LVEF: left ventricular ejection fraction; PA: pulmonary artery PA: Ao: ratio of pulmonary artery to aorta; PEP: pre-ejection period; RVDd: right ventricular diameter measured in diastole; RVDs: right ventricular diameter measured in systole.

### Technique repeatability

Repeatability of technique was excellent for all measurements and time indices (ICC = 0.8-0.96), except for dimensions of the right ventricle (RV), which were good (ICC = 0.64-0.72; see Tables 2 and 3).

### Technique reproducibility

Reproducibility of technique was excellent for the majority of measurements and time indices (ICC = 0.8-0.92), except for FWs and FWd, which were good (ICC = 0.73-0.76) and adequate for those of the RV (ICC = 0.51-0.57; Tables 2 and 3).

### Measurement repeatability

Measurement repeatability for all dimensions and time indices was excellent (ICC = 0.81-0.97; Tables 2 and 3) for both observers and there was minimal variation between measurements (Tables 2 and 3).

### Measurement reproducibility

Measurement reproducibility for dimensions and time indices was excellent for majority of parameters (ICC = 0.80-0.92; Tables 2 and 3), and was good for FWd and RVDd and RVDs (ICC = 0.72-0.80).

### Day effects

There were no differences between measurements and time indices over the three days of examination, except for RVDd (p = 0.01) and RVDs (p < 0.01).

### Animal effects

There were differences in numerous echocardiographic dimensions between species. Pulmonary artery diameter (PA; p < 0.03), Ao (p < 0.04), IVSd (p < 0.01) and FWd (p < 0.02) were smaller in goats than sheep when considering absolute values and when corrected for bodyweight. Pulmonary artery diameter (PA; p = 0.04), IVSd (p = 0.02) and FWd (p = 0.03) were relatively smaller in goats when corrected for bodyweight using Ao. Left atrial diameter (LAD; p = 0.04), IVSs (p = 0.01) and LVDd (p = 0.02) were different between sheep and goats when comparing absolute values, but not when corrected for bodyweight and Ao. Left ventricular diameter in systole (LVDs; p < 0.04) and FWs (p < 0.02) were different between sheep and goats when corrected for bodyweight and using Ao, but not when comparing absolute values. There were no differences for any of the measurements or time indices between the sheep (p = 0.37-0.85) and goats (p = 0.21-0.67) except for FS for both sheep and goats (p < 0.03).

## Discussion

This study has demonstrated that good to excellent technique and measurement reliability can be obtained from echocardiographic dimensions and time indices obtained from sheep and goats, which has not previously been reported. Reliability of these measures becomes important when considering longitudinal studies and clinical evaluation of animals at different time points by different echocardiographers. Reference ranges for internal cardiac structures and time indices were further evaluated for both adult sheep and goats. These data, building on previous studies should improve identification, quantification and assessment of cardiac disease in sheep and goats.

Echocardiography is a practical technique in sheep and goats, although a small acoustic window makes it challenging to achieve some of the images in certain animals, as has been reported previously [7], although was not found to be a problem in a more recent study [8]. The most technically challenging views to obtain were the short axis views of the mitral and aortic valves due to poor visualisation due to the hyperechogenicity of the pleural surface, which is in contrast to a previous study where images of the right ventricular outflow tract and short axis views of the LV for M-mode measurements were more challenging [8]. These differences may relate to slight differences in image acquisition and animal positioning. In this study limbs were abducted rather than lifted and pulled forwards [8].

It has been reported previously in both horses, cattle and goats that ICS width was a limiting factor in the quality of the images obtained [7,23,24]. This was not a problem encountered in this study and one previous one [8] and most likely relates to the small footplate of the phased array probe used compared to a sector probe that was used in the previous study[7]. Majority of the images obtained in this study were good to excellent and there was no correlation between image quality and body size, which in contrast to that proposed in a previous study in goats [8].

To the authors’ knowledge, a full set of internal cardiac dimensions and time indices in adult sheep have not been previously published and time indices have not been published in goats. Internal cardiac dimensions that were statistically different from a previous study in goats [8] included LV dimensions and LVEF. The differences in ventricular dimensions may relate to the difference in bodyweights of the goats used in these two studies or may relate to the reported difficulties of identifying an appropriate level for performing these measurements in the previous study[8]. Right and left ventricular dimensions in sheep were also different when compared to a previous study [2]. These findings may be due to variation of the level at which these measurements were obtained. Improved ultrasonographic technology available for acquisition of images between the current and previous study may also have contributed. It has been previously reported in other species that assessment of RV dimensions is challenging and sensitive to image orientation due to its unusual shape and orientation to the LV [25]. The difference in LVEF in goats when compared to a previous study [8] may relate to the higher heart rates in this study where increased sympathetic tone can affect the degree of contractility or may relate to a different calculation being used to calculate this. It should be noted that the integrated formula of the ultrasound machine for the calculation of the LVEF is a Teichholz-based human-derived algorithm and has not been validated in either sheep or goats. Left atrial dimensions in sheep and goats were not comparable to previous studies [2,7,8] as they were obtained from different views, which require different reference ranges. The current study used a standard technique in horses, whereas previous ones [2,7] used techniques more commonly employed in small animals.

There were numerous differences in cardiac dimensions between sheep and goats in this study. This difference may relate to differences in bodyweight between the sheep and goats used in this study or may be genuine species differences. Many of these differences remained when corrected for bodyweight and cardiac size using Ao. Previous studies performed in horses demonstrate controversy regarding the association between internal cardiac dimensions and bodyweight [18,25,26]. The more recent of these studies [26] supports the findings of an association as has been reported in cattle [27]. Goats have comparatively larger hearts compared to sheep. This may simply be a species difference and may relate to the different thoracic shapes and thus likely cardiac shapes observed in these two species and is described in different breeds of dog [28]. Alternatively it may be due to the fact that goats are adapted to a different environment of living in mountainous, sometimes high altitude regions. Sheep and goats thus require species specific reference ranges for cardiac dimensions.

The variation in dimensions was greater in the current study in goats compared with a previous study [8] and less in sheep that previously reported [2]. As stated above, there is little correlation between bodyweight and cardiac size and therefore although the range of weights was similar in these studies, it may not reflect cardiac size. Also the current study contained far more animals that were unrelated and thus may be providing a more representative range for the population. The dimension differences may also reflect variation in both technique and measurement reliability. Measurement repeatability variation was similar for many parameters in this and a previous study in goats [8], but hasn’t been reported in any other studies in these species.

The time indices assessing cardiac function and one-dimensional distance index (EPSS) were not significantly different between the two groups, which is as would be expected, particularly as the two groups had similar heart rates and fractional shortening. Cardiac valve time indices (ET, PEP) and one-dimensional distance index (EPSS) were similar to previously reported values in cattle [27] and horses [29]. Fractional shortening in the sheep and goats was found to be higher when compared to horses [29], but comparable to cattle [27]. This may be due to this being a more stressful procedure in ruminants compared to horses, although the heart rates were within the normal range for the cattle in the previous study [27] and for the sheep and goats used in this study [30], or may be genuine species differences.

Although none of the animals in this study had audible murmurs, a small number of sheep and goats had evidence of physiological pulmonary, aortic and tricuspid regurgitation. All of these valves appeared structurally normal. Although a previous study in goats described performing CFD [8], these data were not reported. The prevalence of physiological regurgitation was similar to that observed by the authors in cattle undertaken in a previous study, but had a much lower prevalence than reported in Thoroughbred racehorses for all valves [31].

Technique and measurement repeatability was found to be good or excellent for majority of measurements. Standardised views [17,18] were obtained and measurements were performed using recommendations from the American College of Echocardiographers [21] by experienced clinicians who undertook echocardiographic assessments frequently, so this was expected. Measurement repeatability and variation was similar in this study to a previous study in goats [8], although variation in RV and IVSd and FWd were greater than in the current study. This may partly reflect the previously discussed difficulties of imaging the RV. In horses it has been shown that there is greater variation in measures of small structures such as the interventricular septum and free wall as observed to some extent in this study, but more in the previous one [8]. As previously reported [32], measurement repeatability was better than technique repeatability; even with experienced echocardiographers there is more likely to be greater variation in image generation and measurement than acquirement of measurements alone.

There have been no studies assessing reproducibility of technique or measurement in sheep and goats and few in other species [29,33]. One study undertaken in horses demonstrated poor reproducibility in all parameters measured on M-mode echocardiograms of the LV except for the IVSs [33], whereas another equine study found only FS was not reproducible [29]. The current study in sheep and goats found good technique reproducibility, although this was not as good as technique repeatability as would be expected. Reproducibility of technique for the left ventricular free wall was only good and only adequate for RV dimensions. Measurement reproducibility was overall excellent, but only good for free wall and RV dimensions and was not as good as measurement repeatability. Explanations for this are similar to that for variation in dimensions discussed above and similar to that found in one equine study [33]. This good to excellent reproducibility is likely a reflection of the use of standardised views, operator experience and similar training of observers.

Day-to-day differences mainly measure within animal variation [34], although there may also be some effect of machine and measurement technique. This was evaluated in order to assess the likelihood of obtaining similar findings on multiple examinations of the same animal on different days, which is important for longitudinal studies. In this study the only measure affected by day was RV diameter, which is supported by a previous study in goats [8] where variation was minimal except for RV, interventricular and free wall dimensions. Factors affecting day-to-day variation of the measurements may include heart rate [35], technique repeatability and image quality. Although excellent reliability of measurements was obtained in the current study, small differences in measurement may have contributed to the day-to-day variation, particularly as measurements of small structures have been shown to be less repeatable in previous studies [36,37] and RV dimensions are very image orientation dependent [25]. It is unlikely that image quality played a major role since the most of the images were of good to excellent quality and were the only ones evaluated.

An intrinsic problem of the study was that it was not possible to confirm that these animals had normal cardiac structure at post-mortem examination. At 24 month follow-up all sheep and goats used in this study were still alive and had been clinically well.

## Conclusion

This study has demonstrated that using this technique, reliable measurements can be obtained in these species. This means that measurements obtained over time in clinical cases and longitudinal research studies should be comparable. The amount of variation was smaller when evaluating repeatability than reproducibility and smaller for measurement assessment than technique as would be expected. Image acquisition was relatively straightforward and quality of images was good to excellent in the majority obtained. Due to differences between sheep and goats, even when corrected for bodyweight, each species requires its own reference range for cardiac dimensions.

### Endnotes

^a^Aquasonic gel 100, Parker Labs Inc., Fairfield, NJ, USA; ^b^MyLab30, Esoate, Genova, Italy; ^c^GE Ultrasound, Bedford, UK ^d^SPSS for Windows 15.0, SPSS Inc, Chicago, Illinois, USA; ^e^GraphPad 4.0, Graphpad Software, La Jolla, California, USA.

## Abbreviations

2-D: Two-dimensional; ANOVA: Analysis of variance; Ao: Aortic diameter measured in diastole; Ao-cs: Aortic cross-sectional diameter measured in diastole; AoS: Aortic sinus diameter measured in diastole; BCS: Body condition score; bpm: Beats per minute; CFD: Colour-flow Doppler; ECG: Electrocardiogram; EPSS: E-point to septal separation; ET: Ejection time; FS: Fractional shortening; FWd: Left ventricular free wall diameter in diastole; FWs: Left ventricular free wall diameter in systole; ICC: Intra-class correlation coefficient; ICS: Intercostal space; IVSd: Interventricular septal diameter in diastole; IVSs: Interventricular septal diameter in systole; LAD: Left atrial diameter measured in systole; LV: Left ventricular diameter; LVDd: Left ventricular diameter in diastole; LVDs: Left ventricular diameter in systole; LVEF: Left ventricular ejection fraction; PA: Pulmonary artery diameter in diastole; PEP: Pre-ejection period; RV: Right ventricular diameter; RVDd: Right ventricular diameter in diastole; RVDs: Right ventricular diameter in systole; SD: Standard deviation.

## Competing interests

GH’s research scholarship was generously funded by the Horserace Betting Levy Board and TP’s PhD project funded by Department of Environment, Food and Rural Affairs. The author’s have no other completing interests to declare.

## Authors’ contributions

GH and MB conceived the study, all authors were involved in image acquisition and measurement, GH prepared the manuscript and performed all statistical analyses and all authors read and approved the final manuscript.

## Authors’ information

GH is currently Associate Professor in Large Animal Internal Medicine and Critical Care at the University of Nottingham. She is a diplomate of both the American College of Veterinary Internal Medicine and of the American College of Emergency and Critical Care. Additionally she is an Associate Diplomate of the European College of Veterinary Diagnostic Imaging. She completed a large animal internal medicine and critical care residency and a PhD in Equine Cardiology and her research interests include medical imaging in all large animal species. MB is Associate Professor in Veterinary Internal Medicine at the University of Nottingham. He completed an equine medicine residency and a PhD focused on various aspects of equine cardiology and his research interests include many aspects of large animal cardiology. TP is currently in private food animal practice having completed a farm animal residency and PhD in Bovine Respiratory Disease.
